# Interleukin-17A influences the vulnerability rather than the size of established atherosclerotic plaques in apolipoprotein E-deficient mice

**DOI:** 10.1515/biol-2022-0072

**Published:** 2022-09-08

**Authors:** Bo Wang, Xitan Hou, Yaning Sun, Chao Lei, Sha Yang, Yao Zhu, Yingming Jiang, Li Song

**Affiliations:** Institute of Forensic Medicine and Laboratory Medicine, Jining Medical University, Jining City, 272067, Shandong Province, China; Department of Neurology, Affiliated Hospital of Jining Medical University, Jining City, 272067, Shandong Province, China

**Keywords:** IL-17A, IL-17 neutralizing antibody, atherosclerosis, plaque vulnerability, macrophage, efferocytosis

## Abstract

Interleukin (IL)-17A plays a role in the development of atherosclerotic plaques; however, the mechanism remains unclear. In this study, apolipoprotein E-deficient (ApoE^–/–^) mice were fed a high-fat diet to induce atherosclerosis, followed by the treatment with exogenous recombinant IL-17A or the neutralizing antibody to confirm the impact of IL-17A on the established atherosclerotic plaques. We found that both the stimulation of IL-17A and blockage of endogenous IL-17 via antibody did not affect the size of the established plaques. However, IL-17A significantly increased the vulnerability of plaques characterized by the accumulation of lipids and T cells with a concurrent decrease in the number of smooth muscle cells. In addition, the blockage by IL-17 neutralizing antibody attenuated plaque vulnerability. Furthermore, we found that although IL-17A did not affect the efferocytosis of macrophages to apoptotic cells, it promoted the apoptosis of macrophages in the presence of oxidized low-density lipoprotein *in vitro*. Also, IL-17A upregulated chemokines MCP-1 and CXCL-10 expression in the plaques. Our data indicated that IL-17A controlled both SMC and macrophage accumulation and the apoptosis within the plaque, which may further weaken the aorta wall. This study suggests that IL-17A may be a potential therapeutic target for cardiovascular diseases.

## 1 Introduction

Atherosclerosis is the main underlying cause of various cardiovascular and cerebrovascular diseases, typically starting with an inflammatory process. Even though inflammation is a direct result of the human body’s natural healing mechanisms, chronic inflammation has been linked to certain diseases such as heart disease or stroke and may also lead to autoimmune disorders, such as rheumatoid arthritis and lupus. Atherosclerosis is a disease of chronic inflammation characterized by a dysfunctional interplay between the immune apparatus and lipids in blood vessels. Immune cells and nonimmune cells drive plaque inflammation through the complex crosstalk of a large number of inflammatory mediators. The vulnerability of the plaque very much depends on the size and leukocytes as well as multiple cytokine-triggering environments. However, compared to the size, the vulnerability of atherosclerotic plaques is more important in pathogenicity [1]. The vulnerability of the plaques may go deeper on the increased ratio of large lipid necrotic core and inflammatory factors, fewer smooth muscle cells (SMCs), and thinner fibrous caps [2,3]. These vulnerable plaques are more likely to rupture, causing sudden atherothrombosis and vessel occlusion. Numerous cytokines play a vital role in the development of atherosclerosis, increasing plaque vulnerability [4,5].

Interleukin (IL)-17 is a well-studied pro-inflammatory cytokine in various chronic inflammatory diseases [6]. The IL-17 family has six members, labeled IL-17A-F, of which IL-17A is the most studied isoform and is involved in atherosclerosis [7]. However, the precise role of IL-17 in atherogenesis and plaque stability remains unknown [8,9,10,11]. The use of various inhibition techniques, such as neutralization of IL-17 with antibody, creating a genetic deficiency of IL-17, or blocking of IL-17R, has shown variable results [12,13]. Thus, further studies are needed to explore the role of IL-17A in atherosclerosis to evaluate a potential therapeutic strategy targeting IL-17, even though IL-17A is involved in host defense against bacterial infections at the mucosa and involved in autoimmunity diseases. Matrix metalloproteinases (MMPs) from macrophages frequently trigger IL-17A and subsequently upregulate a number of inflammatory factors, some of the adhesion molecules, and increase tendency of apoptosis [11].

Here, we investigated the role of IL-17A in atherosclerotic plaques. With IL-17 and its anti-IL-17 antibody, the possible therapeutic effects are explored to see if IL-17 reduces plaque vulnerability or affects the plaque size. We also detect if IL-17A increases macrophage apoptosis in the presence of oxidized low-density lipoprotein (LDL) or recruits more macrophages into plaques and observe the efferocytosis in plaque sites.

## 2 Materials and methods

### 2.1 Animals

Male ApoE^–/–^ and C57BL/6 mice were procured from the Beijing University and maintained within the animal care facility of Jining Medical University. Next, 8-week-old mice were fed a high-fat diet (HFD, 0.25% cholesterol and 15% cocoa butter) for 12 weeks to induce atherosclerotic plaques. During the last 4 weeks, the mice were administered recombinant IL-17A (IL-17A, 2 μg/mouse, PHC9171; Gibco, NY, USA) or rat anti-mouse IL-17/IL-17A monoclonal neutralizing antibody (IL-17mAb, 50 μg/mouse, MAB421; R&D Systems, Inc., MN, USA) once a week intraperitoneally. Phosphate-buffered saline (PBS) or rat IgG2A (50 μg/mouse; R&D Systems Inc., MN, USA) was used as the control or isotype control, respectively (*n* = 6/group). The mice were humanely sacrificed for the analysis 1 week after the last treatment.


**Ethical approval:** The research related to animal use has been complied with all the relevant national regulations and institutional policies for the care and use of animals. The ethics and review board of Jining Medical University, China approved all animal studies (approval number: 2019-FJ-002).

### 2.2 Measurement of metabolic parameters

Total plasma cholesterol (TCH), triglycerides (TG), and high-density lipoprotein (HDL) were determined using an automated enzymatic technique (7080; Hitachi, Ltd., Japan). LDL was detected with an automated chemically modified technique (Roche Modular DPP System, Roche, Switzerland).

### 2.3 Tissue preparation and staining

The mice were humanely sacrificed and perfused with PBS through the left ventricle. For RNA isolation, the thoracic and abdominal aorta were dissected and treated with the TRIZOL reagent (Invitrogen, Carlsbad, CA, USA). The heart tissues were embedded in the optimal cutting temperature (OCT; Sakura Finetek, Torrance, CA, USA) medium or fixed in a 4% paraformaldehyde solution overnight and then embedded with paraffin. The aortic root, a predilection site for the lesion development in ApoE^–/–^ mice, was serially sectioned from the embedded hearts beginning with the first slide containing all three valves. Serial cryosections of 6–10 µm or paraffin sections of 2–6 µm were dissected longitudinally, and five sections spaced 80 µm apart from each aorta root were stained from each mouse. The cryosections from the aortic root were stained with hematoxylin and eosin (H&E) to detect the size of plaques or with Oil Red O (ORO) stain to detect lipids in the lesions. The frozen sections or paraffin sections were subjected to immunohistochemical staining using rat antimouse macrophage Moma-2 (monocyte/macrophage marker-2) (MCA519G, Bio-Rad Laboratories (Shanghai) Co., Ltd), rabbit antimouse alpha-smooth muscle actin (α-SMA) (ab7817; Abcam Trading (Shanghai) Company Ltd.), and rat antimouse CD (cluster of differentiation) 3 (MCA500GT; Bio-Rad Laboratories (Shanghai) Co., Ltd) antibodies. The TUNEL assay detected apoptosis using the *in situ* cell death detection kit Fluorescein (Roche Diagnostics, Basel, Switzerland). Sirius red staining was performed to detect the collagen levels, and elastic fiber staining (Maxia-bio, Fuzhou, China) was used to detect elastic fibers on paraffin sections. Images were captured by Olympus microscope (IX71; Olympus Corporation, Tokyo, Japan), and computer-assisted histomorphometry was used to quantify the positive staining in the lesions (Image-Pro Plus; Media Cybernetics, Bethesda, MD, USA). Finally, the percentage of the stained area to the total lesion area (%) was calculated. The mean value was calculated from the corresponding three to five consecutive sections from each mouse.

### 2.4 Flow cytometry

The primary macrophage was obtained by injecting sterile 6% soluble starch (1 mL) into the mouse peritoneal cavities. Three days after the injection, cells were isolated from the peritoneal cavity by lavage with cold PBS, and 1 × 10^6^ cells were resuspended in DMEM supplemented with 10% FBS (PBS) and cultured in six-well cell culture plates for 12 h at 37°C in a 5% CO_2_ humidified incubator. Different concentrations of rIL-17A and/or oxidized (ox)-LDL (YB-002; Yiyuan Biotechnologies, Guangzhou, China) were added to the DMEM based on different experimental conditions. Flow cytometry was performed to detect the apoptosis of cells stained with Annexin V-PI kit (Annexin V-FITC [fluorescein isothiocyanate] Apoptosis Detection KIT, Biouniquer, China).

### 2.5 RNA isolation and quantitative real-time PCR

Total RNA was isolated from the thoracic and abdominal aorta using TRIzol reagent (15596-026; Invitrogen, Carlsbad, CA, USA). DNase I was used to remove possible contaminated genomic DNA by 15 min incubation at room temperature, and DNase I was inactivated by adding 1 µL of 25 mM EDTA and heat for 10 min at 65°C. Reverse transcription was performed with RT-PCR quick master mix (PCR-311; TOYOBO, Japan) to obtain cDNA, and quantitative real-time PCR was performed with Ultra SYBR master mixture (CW0956; CW Bio, China) using CFX 96 Real-Time Detection System (Bio-RAD, USA). The threshold cycle (Ct) values, which represent the PCR cycle when a fluorescent signal reaches the threshold, were measured according to the setting of an autocalculated baseline threshold in Bio-Rad CFX Manager software (Bio-Rad, USA). Relative gene expression was calculated by normalizing to the amount of mouse actin gene. The primer sequences and related parameters are presented in Table 1.

**Table 1:** Details and sequences of primers used for RT-qPCR assays

Gene name	Description	Primer sequence (5′–3′)	Amplicon length (bp)	Tm	RT-qPCR efficiency (%)
CXCL-10	C-X-C motif ligand-10	F: CTTAACCACCATCTTCCCAA	152	82.1	98
		R: GATGACACAAGTTCTTCCA			
MCP-1	Monocyte chemoattractant protein-1	F: CCTGCTGTTCACAGTTGCC	229	86.3	95
		R: TGTCTGGACCCATTCCTTCT			
CX3CL1	C-X3-C Motif chemokine ligand 1	F: ACAGACGCTTCTGTGCTGA	211	84.2	93
		R: TCCAAAGCAAGGTCTTCCA			
Beta-actin	Reference gene	F: GGGAAATCGTGCGTGACA	185	86.9	98
		R: CAAGAAGGAAGGCTGGAAAA			

### 2.6 *In vitro* efferocytosis assays

Next, dexamethasone (25 mg/kg) was intraperitoneally injected into 4-week-old C57BL/6 mice to induce the apoptosis of mouse thymocytes. The mice were humanely sacrificed 10 h later, and apoptotic thymocytes were isolated and labeled using carboxyfluorescein succinimidyl ester (CFSE; 21888; Sigma-Aldrich, MO, USA). Next, 5× apoptotic thymocytes were added to macrophages obtained from C57 or ApoE^–/–^ mice and stimulated in the absence or presence of rIL-17A (25 ng/mL). After incubation at 37°C for 60 min, the noningested apoptotic cells were removed by washing with ice-cold PBS. The macrophages were collected and stained with FITC-conjugated F4/80 antibody (ab60343, Abcam Trading (Shanghai) Company Ltd.). A flow cytometry or fluorescent microscopy was used to detect efferocytosis. The index of efferocytosis was calculated as the ratio of FITC^+^ CFSE^+^ cells to all gated cells.

### 2.7 Statistics

Statistical analysis was performed using GraphPad Prism 5.0 software (GraphPad Software, Inc.). Data were expressed as mean ± standard deviation (SD). The unpaired *t*-tests were performed to compare data from different groups. A *P*-value of <0.05 was considered statistically significant.

## 3 Results

### 3.1 IL-17A does not influence the plasma lipid profiles of ApoE^–/–^ mice

Atherosclerosis-prone apolipoprotein E-deficient (Apoe^−/−^) mice display poor lipoprotein clearance with subsequent accumulation of cholesterol ester-enriched particles in the blood, which promote the development of atherosclerotic plaques. There was no significant difference in the body weight and plasma lipid profile, including total cholesterol (TCH), total triglycerides (TG), low-density lipoprotein cholesterol (LDL), and high-density lipoprotein cholesterol (HDL) between the IL-17A group and the control group (Table 2). This observation indicated that the exogenous IL-17A did not influence lipid metabolism in ApoE^–/–^ mice. Table 3 presents that the IL-17 monoclonal neutralizing antibody (IL-17mAb) did not change the weight and lipid profile compared with the isotype group, implying that the effect of IL-17A or IL-17mAb on atherosclerotic plaques was not attributed to the lipid metabolism.

**Table 2:** Exogenous IL-17 treatment did not affect body weight and lipid profile

	Weight (g)	TG (mmol/L)	TCH (mmol/L)	LDL (mmol/L)	HDL (mmol/L)
Control	30.62 ± 0.85	2.40 ± 0.36	25.34 ± 4.2	8.08 ± 1.12	6.61 ± 2.34
IL-17A	30.26 ± 0.78	1.97 ± 0.32	21.66 ± 4.8	10.88 ± 1.02	5.04 ± 0.89
*P*-value	0.76	0.40	0.60	0.11	0.55

The data are represented as mean ± SD. TG – total triglycerides; TCH – total cholesterol; LDL – low-density lipoprotein cholesterol; HDL – high-density lipoprotein cholesterol.

**Table 3:** IL-17 mAb treatment did not affect body weight and lipid profile

	Weight (g)	TG (mmol/L)	TCH (mmol/L)	LDL (mmol/L)	HDL (mmol/L)
Isotype	31.45 ± 0.63	2.46 ± 0.34	27.95 ± 1.29	9.69 ± 0.84	5.42 ± 0.60
IL-17mAb	31.45 ± 0.63	2.20 ± 0.42	24.35 ± 3.35	10.70 ± 0.91	5.39 ± 0.80
*P-*value	0.86	0.66	0.34	0.44	0.98

The data are given as mean ± SD. TG – total triglycerides; TCH – total cholesterol; LDL – low-density lipoprotein cholesterol; HDL – high-density lipoprotein cholesterol.

### 3.2 IL-17A or IL-17mAb did not affect the size of established atherosclerotic plaques in ApoE^–/–^ mice.

**Figure 1:**
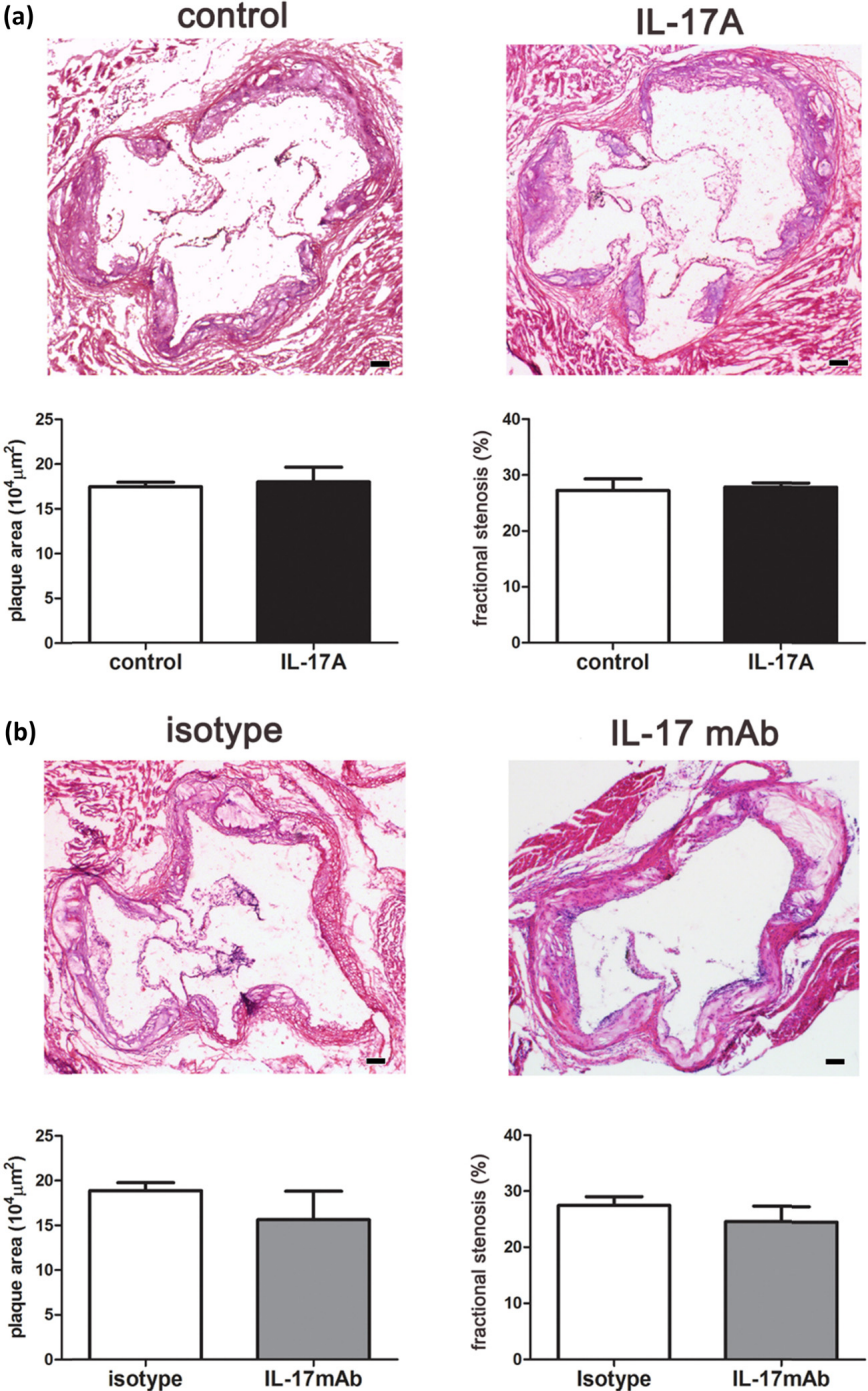
IL-17A or IL-17mAb did not affect the size of atherosclerotic plaques in ApoE^–/–^ mice. (a) H&E stain of aortic root sections shows the atherosclerotic plaques from the control and IL-17A groups (20× magnification). The plaque area (µm^2^) represents the sum of all areas occupied by plaques in every pathological section. Fractional stenosis (%) represents the ratio of total plaque area to the cross-sectional area of the aortic root. (b) H&E stain of aortic root sections shows the size of atherosclerotic plaques from isotype control and IL-17mAb groups (20× magnification). The plaque area (µm^2^) represents the sum of all areas occupied by plaques in every pathological section. Fractional stenosis (%) represents the ratio of total plaque area to the cross-sectional area of the aortic root. Data are shown as mean ± SD, scale bar = 100 µm, *n* = 6.

The ApoE^–/–^ mice were fed with a HFD for 8 weeks to induce atherosclerotic plaques, followed by the treatment with recombinant IL-17A (2 μg/mouse) once a week for 4 weeks (IL-17A group). The control group received the same volume of PBS following the same pattern. The H&E staining showed that IL-17A did not affect the size of the established plaques. Next, we measured the ratio of total plaque area to aortic root cross-sectional area to assess the fractional stenosis of vascular lumen and found that IL-17A treatment did not change this parameter (Figure 1a). The same models were treated with an anti-IL-17A monoclonal neutralizing antibody (IL-17mAb group) to confirm the role of IL-17A in atherosclerosis. We found that IL-17mAb also did not change the size and the fractional stenosis of established plaques compared with the isotype control (Figure 1b).

### 3.3 IL-17A increased the vulnerability of the established plaques

**Figure 2:**
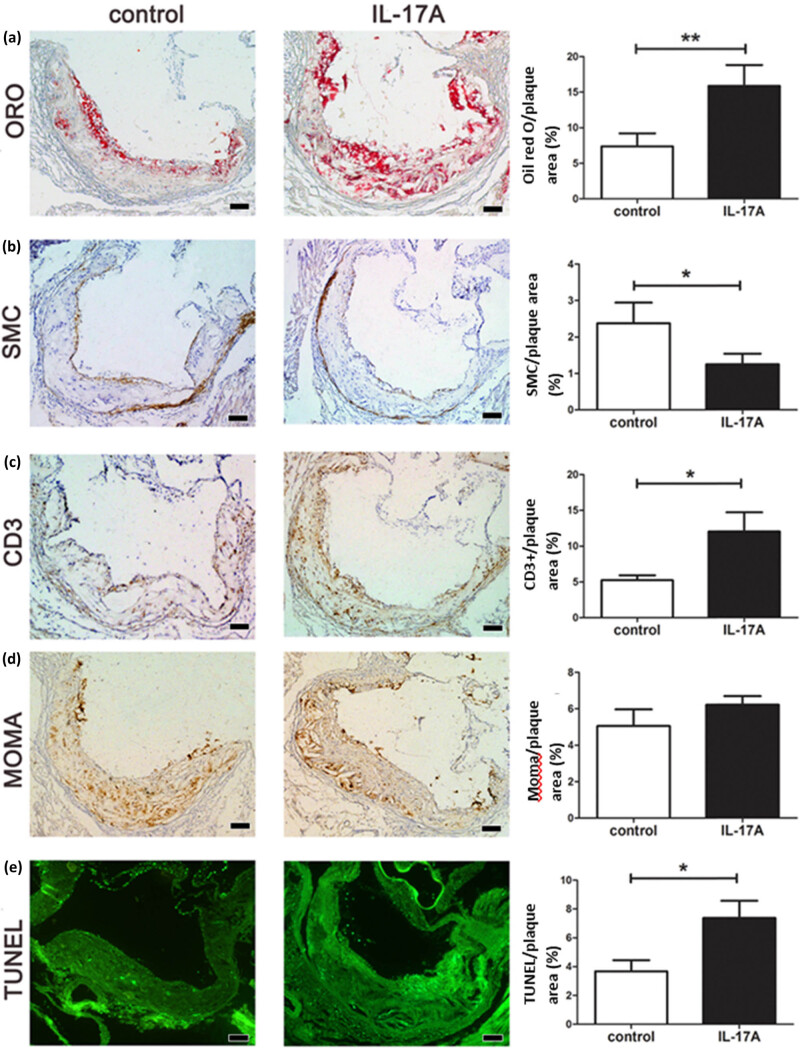
IL-17A increased the vulnerability of the aortic wall in the established plaques in ApoE^–/–^ mice by immunohistochemistry and TUNEL analysis: microscopic image and histomorphometry value. (a) Lipids in the aortic wall via ORO stain; (b) SMCs of immunohistochemistry stain by α-SMA; (c) CD3^+^ T cells of immunohistochemistry stain; (d) macrophages of MOMA immunohistochemistry stain; and (e) apoptosis of TUNEL stain from the control and IL-17A groups (40× magnification). The proportion of positive staining area to the total plaque area (%) was calculated and plotted by comparison between groups. Data are shown as mean ± SD. Scale bar = 100 µm, *n* = 6, *P < 0.05.

The vulnerability of plaques is more critical than the size in the development of atherosclerosis-related diseases. We tested the histomorphometry indexes related to the vulnerability and found that IL-17A changed the composition of established plaques, characterized by the significant accumulation of lipids (Figure 2a) and the decrease in SMC in the local lesions (Figure 2b). In addition, there was a significant increase in the infiltration of CD3^+^ T cells (Figure 2c) and the TUNEL+ apoptotic cells in lesions of the IL-17A group compared with the control group (Figure 2e). Although there was no difference in the MOMA-2^+^ macrophage content (Figure 2d), collagen, and elastic fibers (data not shown) between the IL-17A and control group, it suggested that IL-17A treatment promoted the vulnerability of the established plaques based on various indicators.

### 3.4 IL-17mAb attenuated the vulnerability of atherosclerotic plaques in ApoE^–/–^ mice

**Figure 3:**
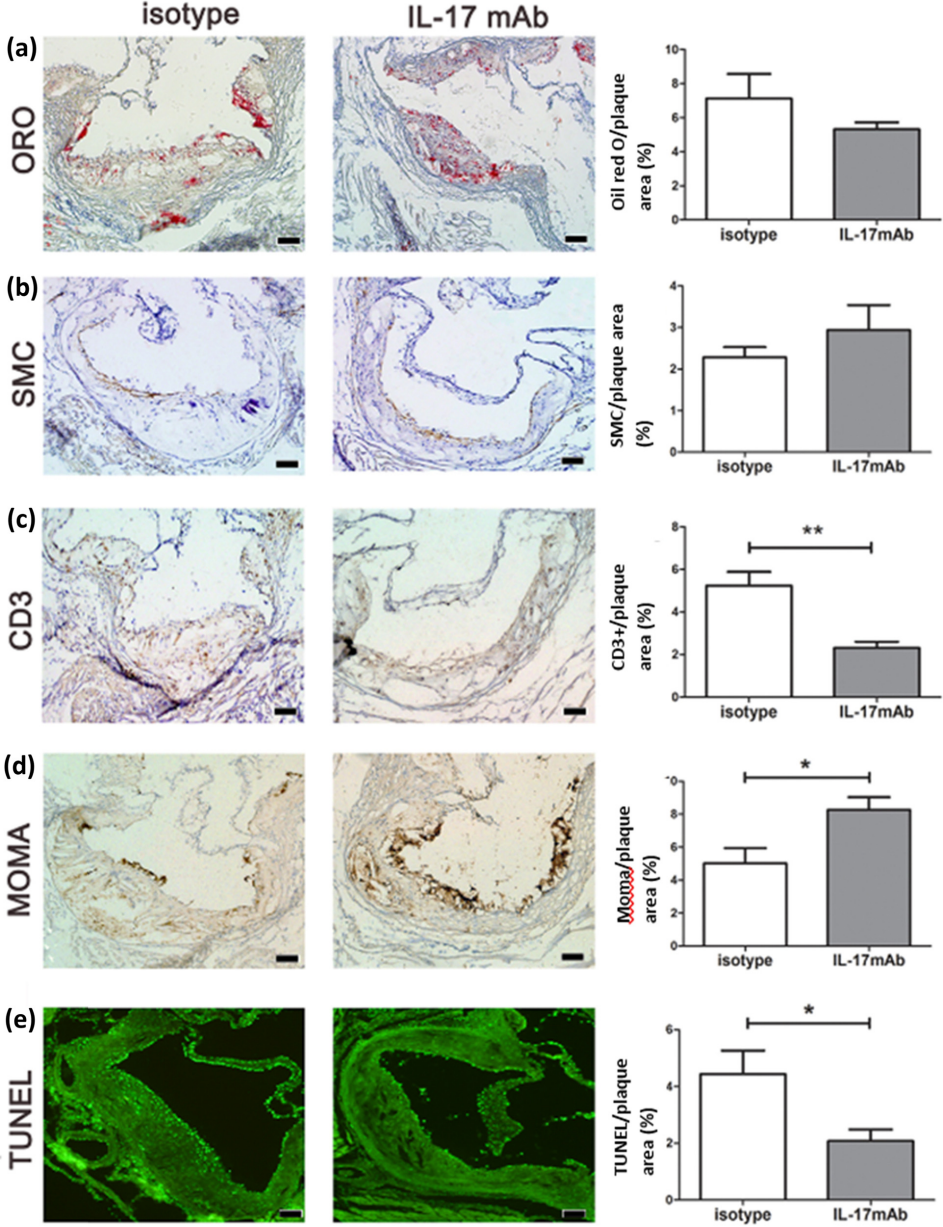
IL-17mAb attenuated the vulnerability of plaques in ApoE^–/–^ mice by immunohistochemistry and TUNEL analysis: Microscopic image and histomorphometry value. (a) Lipid via ORO stain; (b) SMCs of immunohistochemistry stain; (c) CD3^+^ T cells of immunohistochemistry stain; (d) macrophages of MOMA immunohistochemistry stain from the isotype and IL-17mAb groups (40× magnification); (e) apoptosis of TUNEL stain. The proportion of positive staining area to the total plaque area (%) was calculated and plotted by comparison between groups. Data are shown as mean ± SD. Scale bar = 100 µm, *n* = 6, *P < 0.05; **P < 0.01.

Next, the mouse models were treated with anti-IL-17A monoclonal neutralizing antibody (IL-17mAb) to further confirm the impact of IL-17A on atherosclerotic plaques and evaluate for possible therapeutic approaches. We found that IL-17mAb significantly decreased the content of CD3^+^ T cells (Figure 3c) and TUNEL+ apoptotic cells (Figure 3d) compared with the isotype control. IL-17mAb also decreased the lipid levels (Figure 3a) and increased the content of SMC (Figure 3b) although the difference was not statistically significant. There was no change in the collagen and elastic fiber content in the IL-17mAb group compared with the isotype control group (data not shown). Interestingly, we observed that IL-17mAb markedly elevated the macrophage content in the local lesions (Figure 3e). In summary, we believe that the blockade of IL-17A using neutralizing antibody can attenuate the vulnerability of atherosclerotic plaques.

### 3.5 IL-17A promoted both macrophage apoptosis and macrophage chemokines

**Figure 4:**
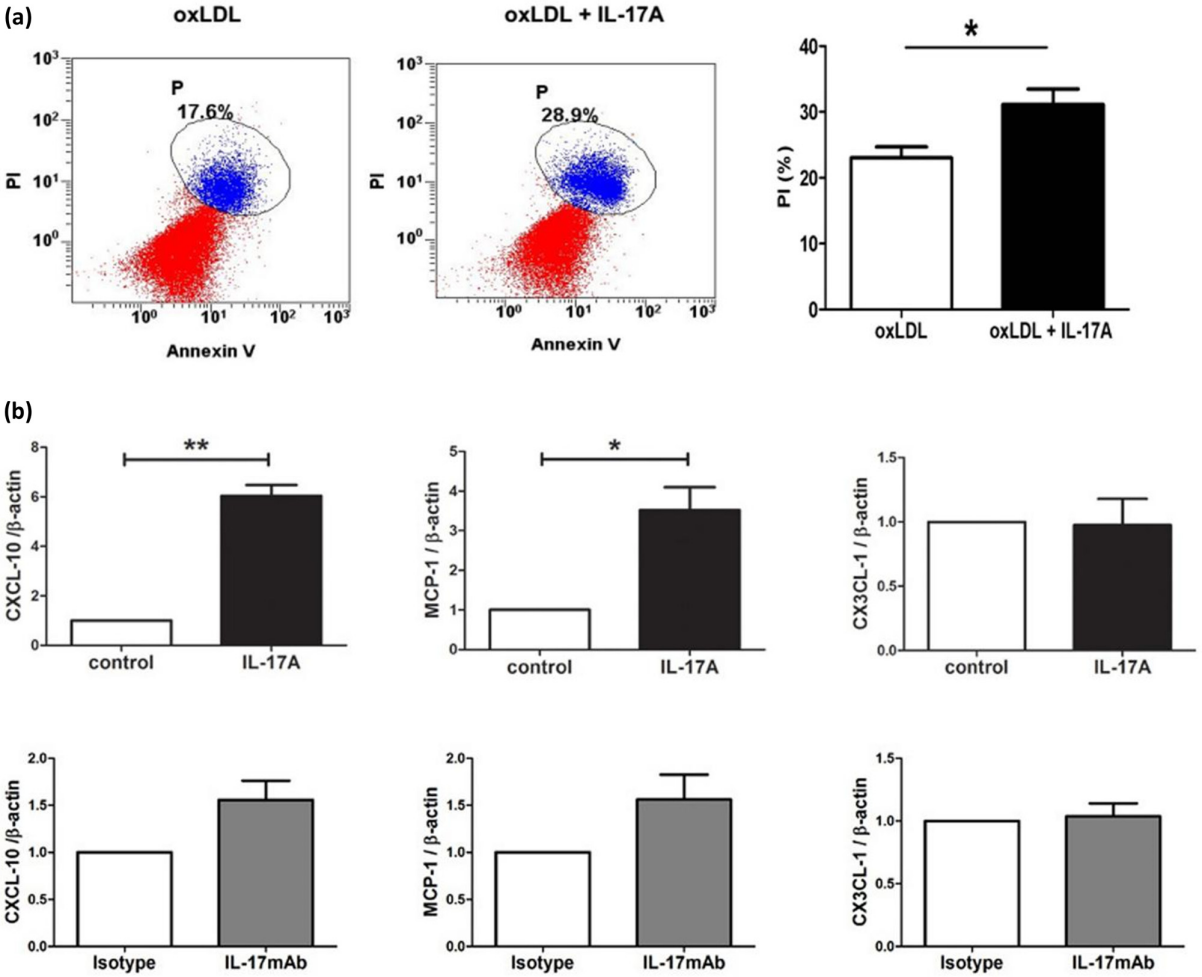
IL-17A promoted both macrophage apoptosis and macrophage chemokines. (a) IL-17A promoted the apoptosis of macrophages in the presence of ox-LDL. Flow cytometry was used to detect the apoptosis of cells, which were stained using Annexin V-PI kit. Three independent experiments were performed. *P < 0.05. (b) The expression of chemokine mRNAs in the thoracic and abdominal aorta between different groups was quantified by RT-PCR analysis. *P < 0.05 and **P < 0.01.

To explore the mechanism of IL-17A on atherosclerotic plaques, we tested the effect of IL-17A on macrophages *in vitro* and found that IL-17A increased the apoptosis of macrophages in the presence of oxidized LDL (ox-LDL; Figure 4a). In addition, we found that IL-17A increased the mRNA expression of macrophage chemokines, including CXCL-10 and MCP-1, in the arterial wall (Figure 4b), which implied that more macrophages were recruited into the plaques. It suggested that more macrophages migrated to the local lesions and died under the action of IL-17A, resulting in the constant levels of macrophages in the IL-17A group compared with the control group. This study further showed that IL-17mAb treatment did not change the level of chemokines compared with the isotype control group (Figure 4b). It suggested that the increase in macrophages in the IL-17mAb group (Figure 3e) can be attributed to a probable protective effect of IL-17mAb on the death of macrophages.

### 3.6 IL-17A did not affect the efferocytosis of macrophages.

**Figure 5:**
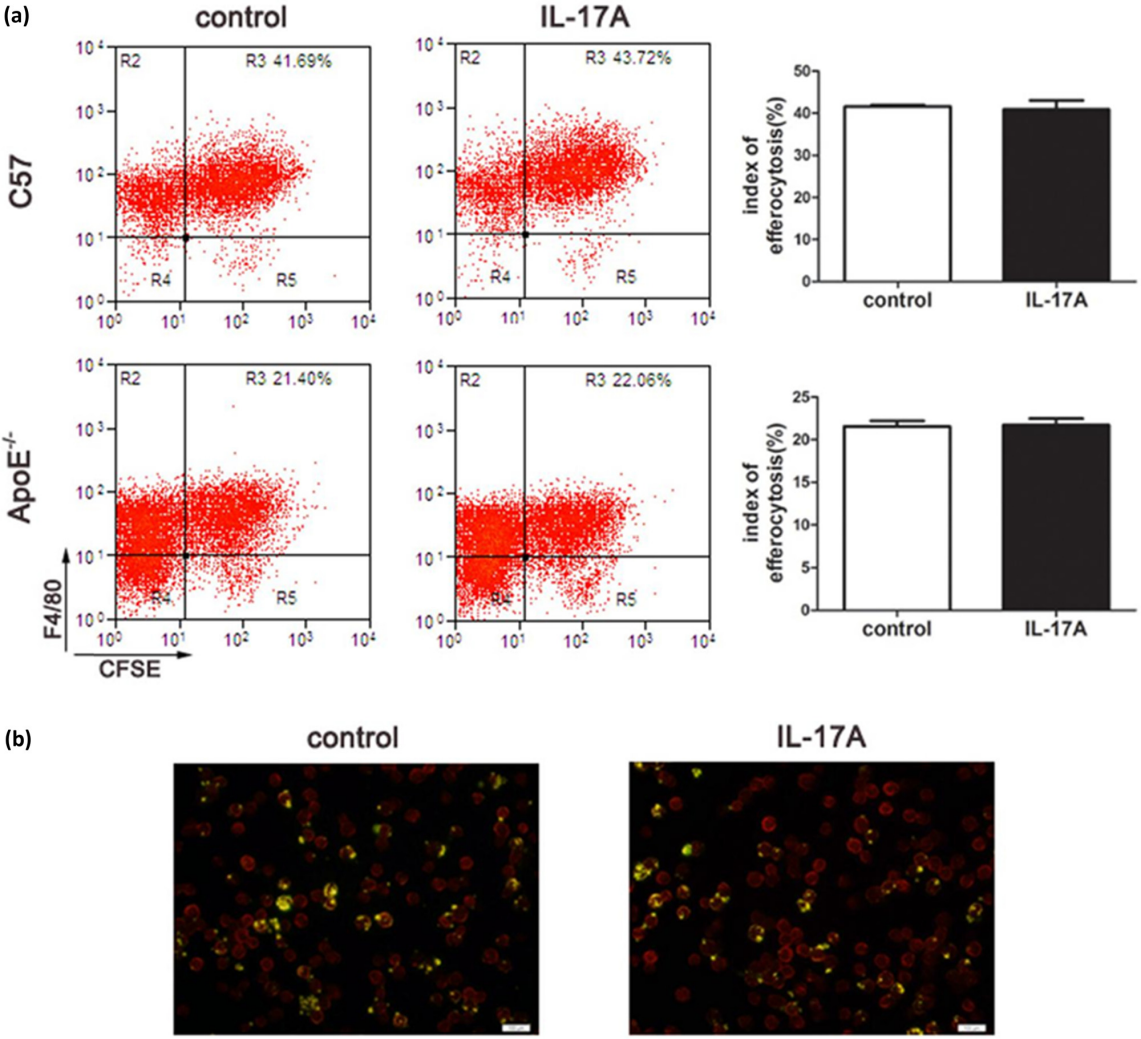
IL-17A did not affect the efferocytosis of macrophages. (a) Flow cytometry was used to analyze the efferocytosis of macrophages isolated from C57BL/6 J or ApoE^–/–^ mice. (b) Fluorescent staining of the efferocytosis of macrophage isolated from C57BL/6 J mice. The mouse apoptotic thymocytes were stained with CFSE, and the macrophage was stained with FITC-F4/80 antibodies. The index of efferocytosis was calculated by comparing double-positive cells with total cells. The experiments were repeated six times. Scale of bar = 100 µm.

Efferocytosis is one fundamental function of macrophages that is for the clearance of dying cells or debris; here, the efferocytosis is mainly on apoptotic cells. It has been reported that IL-17A affects the efferocytosis by macrophage phagocytosis of apoptotic cells [14]. Thus, we tested the effect of IL-17A on the macrophage phagocytosis of apoptotic cells by labeling C57. However, the flow cytometry and fluorescence microscopy results showed that IL-17A had no influence on the index of efferocytosis of macrophages from either C57 or ApoE^–/–^ mice (Figure 5).

## 4 Discussion

It is widely demonstrated that IL-17A induces macrophages in inflammatory sites, which play a scavenger role, but more notably trigger the plaque and plaque ruptures, clinic atherosclerotic lesions, or thrombosis. This is probably one of the double effective mechanisms in inflammation. At the site of plaque, one of the processes is involved with lipoprotein ingestion and accumulation giving rise to foam cells filled with different leukocytes, but particularly macrophages. Accumulation of foam cells particularly contributes to plaque formation and rupture as lipid storage increases. Clinical trials have demonstrated that IL-17A has a stronger association with plaque vulnerability and cardiovascular diseases compared with other cytokines [15,16,17]. This is particularly suggested by a recent study that IL-17 did not contribute to the development of early atherosclerosis [18]. Hereby, we investigated the impact of IL-17A on the established plaques and found that IL-17A did not affect the size but increased the vulnerability of plaques by decreasing the number of SMC and increasing lipids, TUNEL+ apoptosis, and CD3^+^ T cells through the analysis of histology, immunohistochemistry, flow cytometry, and efferocytosis. These data confirmed the impact of IL-17 on the vulnerability of plaques. However, IL-17A did not affect all parameters of atherosclerotic plaques. We did not observe any changes in macrophages, collagen, or elastic fibers in the IL-17A group compared with the control group. Even though the differential analysis of the impact of IL-17 on atherosclerotic plaques could be drawn by adopting different indexes, which could partially explain the varying results from different studies.

In the present research, we found that IL-17A reduced the plaque stability instead of changing the size of the established plaques. This is because anti-IL-17A antibody significantly reduced the content of CD3^+^ T cells and apoptotic cells compared with the isotype control, which suggested that the blockage of IL-17A could partially attenuate the vulnerability of the established plaques. This result was similar to some studies [19,20] and also different from others [21]. Nonetheless, the application of the IL-17A antibody did not significantly affect the size of the plaques, implying that the interruption of IL-17A might have a stronger impact on the vulnerability than the size of the established plaques.

We investigated the effect of IL-17A on macrophages to explore the mechanism of IL-17A on atherosclerotic plaques. We found that IL-17A upregulated the apoptosis of macrophages in the presence of ox-LDL. Meanwhile, we found that IL-17A increased the mRNA levels of macrophage chemokines, including CXCL-10 and MCP-1, in the arterial wall, indicating that IL-17A recruited more macrophages to the lesions, thus inducing their apoptosis. We tested the effect of IL-17A on the ability of macrophages to clean up the apoptotic cells, namely, efferocytosis, and found that IL-17A did not influence the scavenging. The results of efferocytosis were different from the other studies [14], which were probably due to the presence of different target cells selected for phagocytosis analysis. Here, we applied apoptotic thymocytes as target cells instead of aged neutrophils or latex beads that were used in the study by Silverpil et al. [14]. It is reported that IL-17 also increased the apoptosis of SMC [22], which may partly indicate the decrease in SMC in the IL-17A group in this study. The increase in apoptotic macrophages and SMC could induce secondary inflammation presented as increased CD3^+^ T cells. In addition, the effect of increased chemokines, such as CXCL-10 and MCP-1, recruited more T cells into local lesions [23,24,25]. In the IL-17mAb group, we observed a marked elevation in the number of macrophages, possibly resulting from the protective role of IL-17mAb in macrophage deaths in the presence of ox-LDL. The increased macrophages might clean up more apoptosis cells in the IL-17mAb group, which induced less secondary inflammation characterized by decreased CD3^+^ T cells.

There are reports that IL-17A antibody has been successfully used for psoriasis, an autoimmunity disease, but related to CVD as well. Ixekizumab is a humanized IgG4 IL-17A monoclonal antibody, and secukinumab is a fully human IgG1 IL-17A monoclonal antibody, both highly effective in treating patients with moderate-to-severe plaque psoriasis in three phases of clinical trials [26]. IL-17A antibody also improved cardiovascular state for these patients; however, not much data are available about the risk reducing of atherosclerotic plaques yet. Interestingly, the IL-17A inhibition of secukinumab and ixekizumab was recently tentatively used in Covid-19 patients in China even though the effectivity has not been fully evaluated [27].

**Figure 6:**
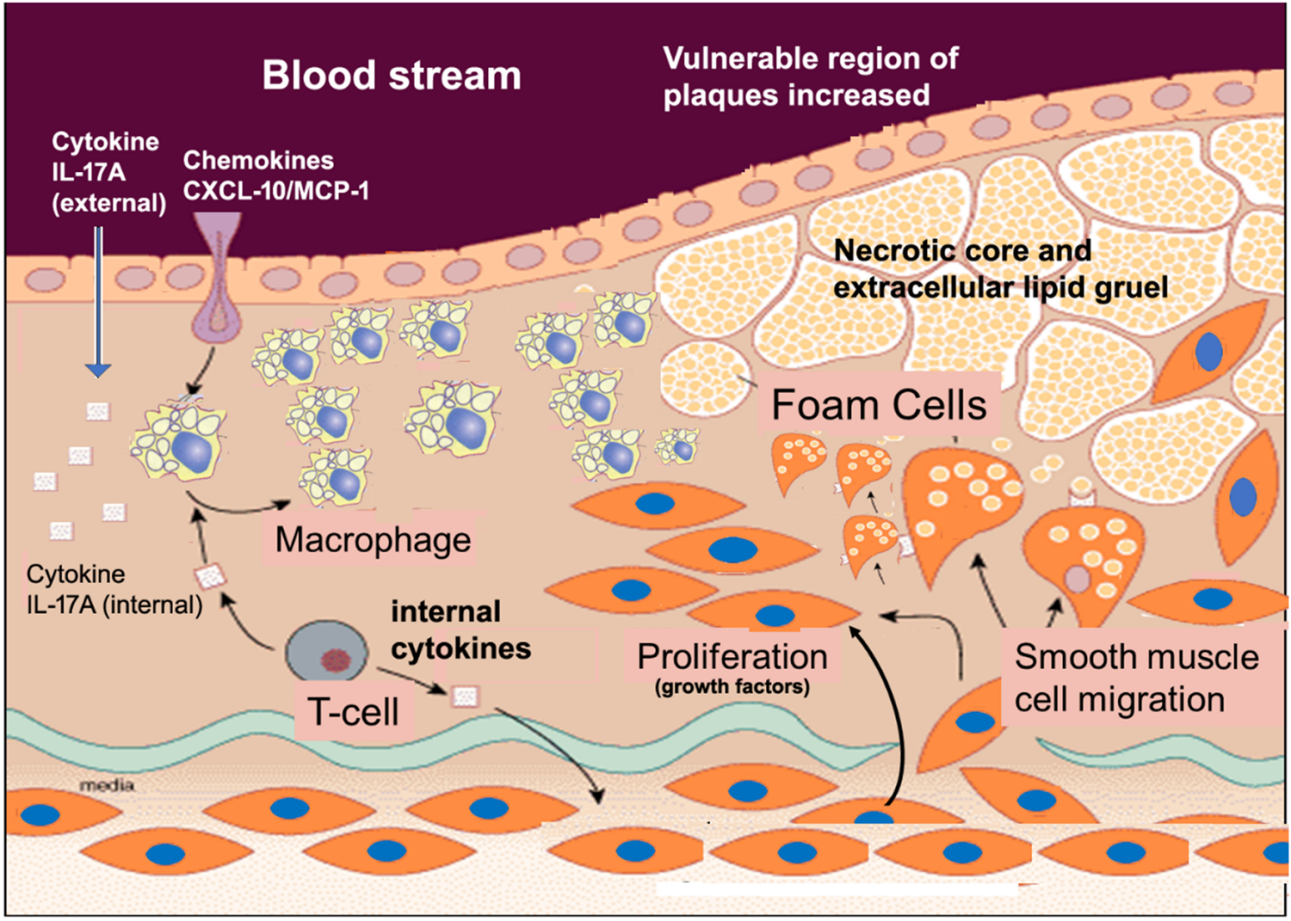
A hypothetic signaling that IL-17A is associated with CXCL-10/MCP-1 in the participation for recruiting and activating macrophages, and triggers the growth and migration of SMCs, which gradually take on necrotic core and extracellular lipid gruels to form the foam cell, a precursor of plaques. IL-17A signaling may increase the vulnerability of atherosclerotic plaques.

In summary, despite the intrigue of the association of chemokines with macrophages, we demonstrated that IL-17A influenced the vulnerability instead of the size of the established plaques of ApoE^–/–^ mice. IL-17A increased the apoptosis of macrophages instead of impacting the efferocytosis of macrophages, which is a common cause of plaques vulnerability. We proved that IL-17mAb partly alleviated plaque vulnerability, mainly manifested in reducing inflammation and apoptosis, providing a new strategy for studying atherosclerosis (Figure 6).
